# Interactive web-based mapping: bridging technology and data for health

**DOI:** 10.1186/1476-072X-10-69

**Published:** 2011-12-23

**Authors:** Linda Highfield, Jutas Arthasarnprasit, Cecelia A Ottenweller, Arnaud Dasprez

**Affiliations:** 1 St. Luke's Episcopal Health Charities, Houston, TX, USA; 2 Texas A&M School of Rural Public Health, Department of Epidemiology and Biostatistics, College Station, TX, USA; 3 Hexagroup LLC, Houston, TX, USA

**Keywords:** interactive mapping, health disparities, geographic information systems

## Abstract

**Background:**

The Community Health Information System (CHIS) online mapping system was first launched in 1998. Its overarching goal was to provide researchers, residents and organizations access to health related data reflecting the overall health and well-being of their communities within the Greater Houston area. In September 2009, initial planning and development began for the next generation of CHIS. The overarching goal for the new version remained to make health data easily accessible for a wide variety of research audiences. However, in the new version we specifically sought to make the CHIS truly interactive and give the user more control over data selection and reporting.

**Results:**

In July 2011, a beta version of the next-generation of the application was launched. This next-generation is also a web based interactive mapping tool comprised of two distinct portals: the Breast Health Portal and Project Safety Net. Both are accessed via a Google mapping interface. Geographic coverage for the portals is currently an 8 county region centered on Harris County, Texas. Data accessed by the application include Census 2000, Census 2010 (underway), cancer incidence from the Texas Cancer Registry (TX Dept. of State Health Services), death data from Texas Vital Statistics, clinic locations for free and low-cost health services, along with service lists, hours of operation, payment options and languages spoken, uninsured and poverty data.

**Conclusions:**

The system features query on the fly technology, which means the data is not generated until the query is provided to the system. This allows users to interact in real-time with the databases and generate customized reports and maps. To the author's knowledge, the Breast Health Portal and Project Safety Net are the first local-scale interactive online mapping interfaces for public health data which allow users to control the data generated. For example, users may generate breast cancer incidence rates by Census tract, in real time, for women aged 40-64. Conversely, they could then generate the same rates for women aged 35-55. The queries are user controlled.

## Introduction

Geospatial assessment and communication of disease patterns, risk and health outcomes, has historically been limited to specialized researchers, using specialized tools [1]. With the advent of web 2.0, the landscape of accessing health information has shifted, and more researchers (academic and non-academic) are using the web for data sharing, research and planning purposes [2]. Many health departments now provide public access to their health statistics via the Internet, including morbidity and mortality indicators [3]. This has promoted user involvement and data examination beyond the traditional academic model [3,4]. The web can serve as a tool for sharing real-time information across a spectrum of potential users; additionally geographic information systems (GIS) technologies on the web have become increasingly popular and user- friendly.

GIS technologies are tools that allow for the storage, management, manipulation and visualization of spatial data [5]. Web-based GIS has the potential to connect a large number of researchers and lay people with geospatial public health data. Distributing and sharing geospatial data on the web can aid health planners, policy researchers and decision-makers in their collaborative efforts to improve public health outcomes and design interventions targeted to local populations [5]. At the forefront of this movement to share data across the spectrum was the Community Health Information System (CHIS) online mapping system, which was first launched in 1998. Its overarching goal was to provide researchers, residents and organizations access to health related data reflecting the overall health and well-being of their communities within the Greater Houston Area. The central theme for the development of the original platform was to create a tool that could be used to visualize, analyze and ultimately reduce health disparities. The original interface effectively connected a wide audience with information vital to developing action plans to address identified health needs in underserved areas.

However, over time, a number of issues were identified that limited the utility of the system. The original design of the CHIS mapping system required the download of an ActiveX control, which is a Microsoft framework that allows programmers to take advantage of additional features that would not normally be available on the browser. It also only worked in Internet Explorer, limiting user accessibility as a host of new browsers came online. The original CHIS provided users pre-categorized static maps, which could be overlaid with clinic locations. Additionally, users could access stand-alone reports containing additional data about the area of interest, such as Census data on population.

As the CHIS was developed and grew over time, the back-end database for the geo-referenced data became an issue. Data was loaded into multiple tables, sometimes containing repetitive information, due to the nature of the piecemeal addition of new data. Maintaining up-to-date clinic information is vital to the success of the portals, therefore improving usability in the back-end of the application for both the clinics uploading information and the administrator was important. And the original multiple databases housing all of the data needed to be analyzed and synthesized into a single database that was flexible enough to expand for future data types. These issues are not unique to CHIS. Models developed over time tend to run into issues with data storage and must be adaptive to continue to serve their target audience.

A number of online public health mapping tools have been developed since the launch of CHIS in 1998, including many developed by State and local public health departments. Almost all of these systems are interactive, allowing the user to query data and then generate a map [6]. One of the current limitations with these systems is an issue of scale. Most of these systems provide data at a resolution no smaller than County level. Those providing sub-County data, to date, only do so in a pre-categorized static map or as separate reports given in conjunction with the County- level data. None, to the authors knowledge, provide both user-generated local-scale geographical and attribute data (e.g., a resolution smaller than County, such as Census tract, block or Zip code). Local-scale geographical and attribute data is critical for conducting research, targeting interventions and siting new service locations. Many public health research and intervention projects are intended to affect a population at a scale smaller than County resolution. Even still, data that is local-scale is typically provided in an aggregated form, such as Census tract, presenting limitations on the utility of the data. Researchers, planners and service providers need to be aware of the issues surrounding scale and data aggregation, such as the modifiable areal unit problem [7]. The Florida CHARTS is an example of an online interactive health GIS tool providing both County and sub-County information [8]. This site provides the user with the ability to query data on birth or death rates by County across various time periods. Additionally, users can select Census Tracts to open a separate report containing demographic data for the selected tract. While current implementations, such as CHARTS, serve as an important data and planning resource, they do not provide the end-user with the ability to generate customized local-scale maps and reports based on specific user-driven criteria in real-time. The ability to generate data at a meaningful level of spatial aggregation has been cited as a major challenge to overcome with interactive mapping systems in public health [9].

Technological advances have made it possible to begin addressing this challenge, including Google Earth and the Google Maps API launched in 2005. These tools followed the widespread adoption of Google as a web search engine in the early 2000's. The Google Data APIs allow programmers to create applications that read and write data from the Google service [10]. The widespread use of Google technologies has created a large number of potential users who already have knowledge of how to use these tools. Additionally, these technologies can easily be used to create custom applications in an interface that appears seamless to the user [5,11-16].

Leveraging the power of Google API technologies, we set out to create a local-scale interactive web-based system for public health data with the next generation of CHIS.

The overarching goal for the new version of the system was to make health data easily accessible for a wide variety of research audiences with an interest in developing health programs benefiting under-served populations such as academic researchers, policy makers, community planners and non-profits who conduct research. This information can be used in grant applications. Specifically, the goal was to create a system in which researchers could easily access demographic, health related measures, clinic location, and clinic service data. The objective was to provide a user-friendly tool to better inform public health intervention development, healthcare expansion and service delivery and, most importantly improve access to care and reduce health disparities in the Greater Houston region. To achieve these goals and objectives, an online, real-time interactive local-scale database system was created.

## Results

**Figure 1:**
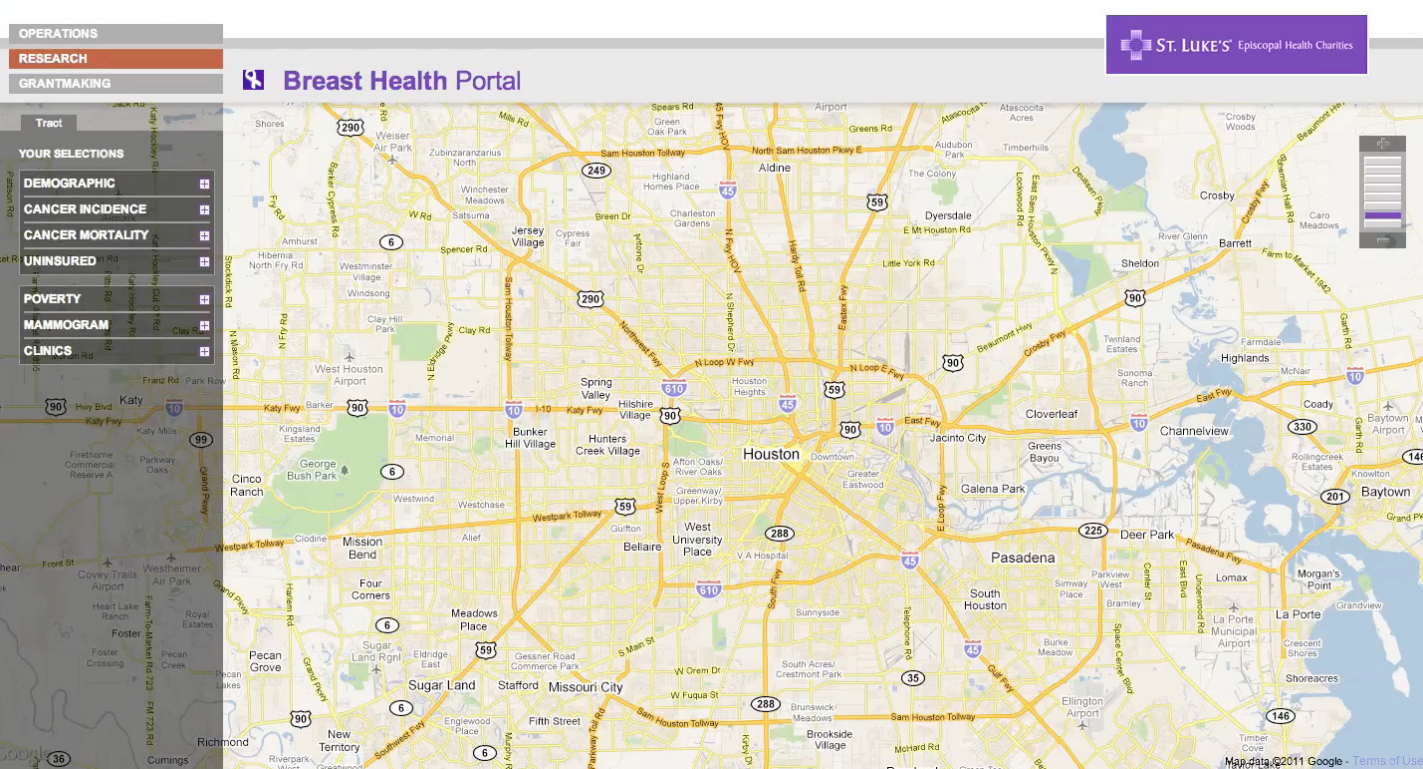
**Landing page for the Breast Health Portal**.

**Figure 2:**
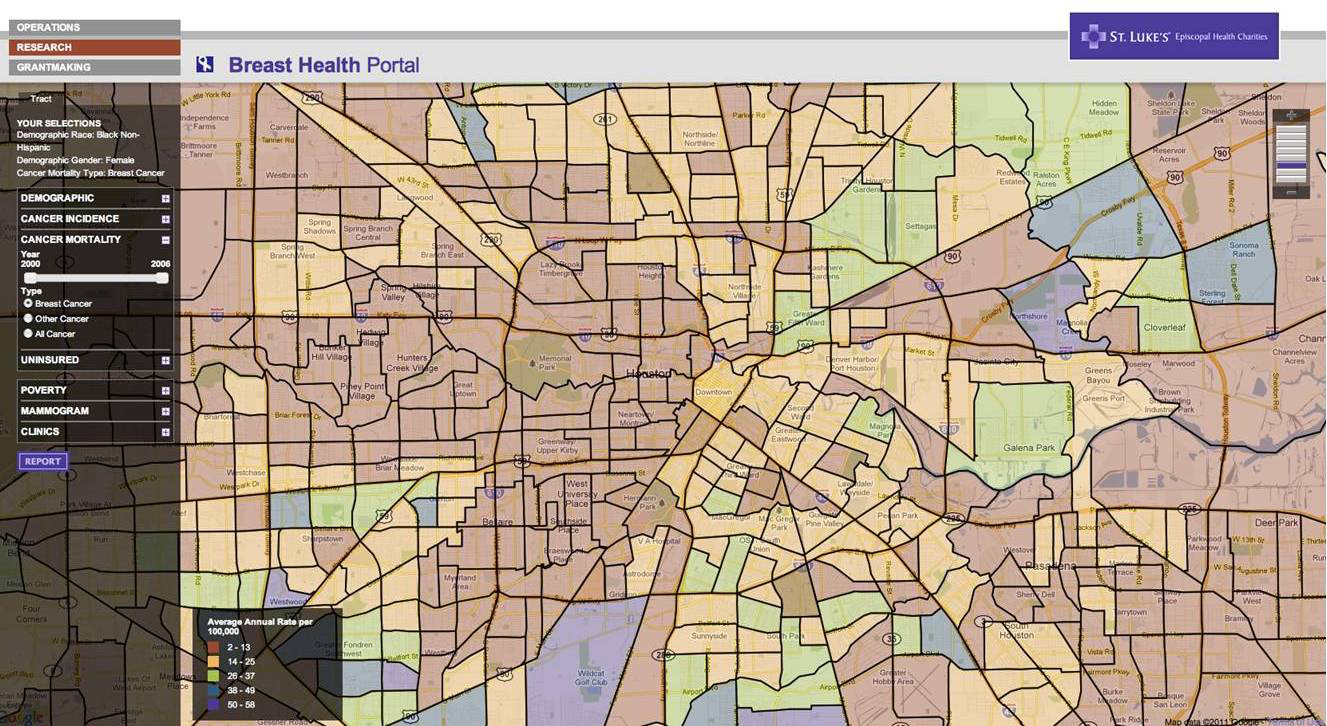
**Example of an interactive query in the Breast Health Portal**.

**Figure 3:**
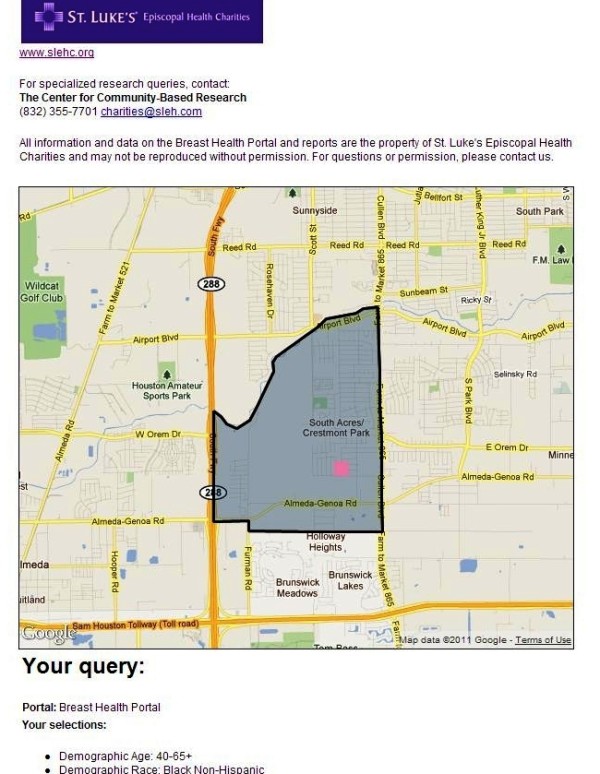
**Example of Breast Health Portal report function**.

The Beta sites for both the BHP and PSN are functional and available for review online. The BHP can be accessed at: http://interactive-mapping.slehc.org/breast-health-portal/census.aspx. The PSN can be accessed at: http://interactive-mapping.slehc.org/project-safety-net/census.aspx. The landing page for the BHP is shown in Figure 1. Both portals allow the user to zoom in and out and pan using standard Google navigation tools (Figure 1). Both portals provide data on rates of disease, including cancer incidence and mortality and the top five leading causes of death as defined by the Centers for Disease Prevention and Control. Due to data instability, areas with less than 20 cases or deaths in the selected time period were suppressed. An example of an interactive query is shown in Figure 2; the report function is shown in Figure 3. To date, the response to the Beta portals has been very positive. Over a two month period, following the launch of the beta site, a total of 395 visits by 285 unique visitors were reported. The average visit length was just over two minutes.

## Discussion

The portals present users with an easily accessible platform for accessing data, addressing research questions and service delivery decisions. For example, a Federally Qualified Health Center (FQHC) in the local area used the data on PSN related to medically underserved areas, the uninsured, population demographics and clinic locations to determine the proposed site for a new clinic (personal communication). Researchers interested in studying the relationship between mammography screening availability and uninsured women of screening age have used the BHP to address the relationship between these variables. These are two of many examples of how the system can be utilized to benefit researchers, planners and non-profits in the area covered by the portal.

The creation of the next generation of the portals had to be completed given a number of constraints: The portals needed to be accessible to as many people as possible, therefore the technology used to access them needed to be universally accessible, low cost, would not require any additional downloads and be as browser-agnostic as possible. The portals also needed to be as interactive as possible, allowing users to dynamically define their research parameters rather than being forced to accept preset queries.

Google technologies, including Google mapping, were chosen for a variety of reasons: First, Google and Google Maps are well known and widely used by a cross-section of the portals' target audience, which means reduced barriers to use. Compatibility was also a powerful selection criterion; no third-party implementation was necessary to install and access the maps. Google maps were a low-cost choice, since they were free and supported by SSL protocol when we began development of the Beta portals. Google announced usage limits to the Maps API in October, 2011 For more details, see http://googlegeodevelopers.blogspot.com/2011/10/introduction-of-usage-limits-to-maps.html. As can be seen in the article, Google intends the charges to affect power-users of the API. No charges are incurred with less than 25,000 map loads per day per API. The SLEHC portals are not expected to exceed that limit.

Google also provides an ideal technology for mobile support with access on Google Android-enabled devices and Apple ios devices (iPhone and iPad). Opportunities to integrate with future Google-developed innovative tools, such as Google Public Data Explorer, were important. We plan to enable the portals for mobile access and use Google Public Data Explorer in the next version of the system. Utilizing a variety of technologies in the Beta portals allowed users to interact with and query local-scale health related data in real time. Nevertheless, four core challenges to the technical implementation had to be overcome: 1) Polygon loading time, 2) Data consolidation and standardization, 3) Application usability, and 4) Real-time data calculation.

### Polygon loading time

There were 4404 polygons of Census tracts and 2884 Zip code polygons to load in the user's browser. If they loaded simultaneously it would have made the user experience unacceptably slow. Our solution was to constrain loading to the given viewing area. We detected the coordinates of the upper left and lower right corners of the display window and then displayed/calculated only those polygons within the proscribed area. This significantly increased the speed of the system and improved the user experience by reducing wait time for results. The system currently takes just under 1 second to load each polygon.

### Consolidation and standardization of data

The first generation of the application (CHIS) used data from 12 separate databases. To streamline the data and improve functionality for the next iteration, the databases were consolidated into a single database, requiring a massive reorganization. A majority of the data definition and field types in the database were stored in order to allow scalability. We also strictly defined additional data definition tables that will allow the system administrator to configure the data fields for any future data provided by non-federal sources and restricted data format to comma-separated variable (CSV), which significantly improved importation of data into the system and allowed data to go live immediately after upload (assuming the data are checked and clean).

### Usability, interactivity, presentation

One of the constraints of the redevelopment of the system specified interactive, user-defined queries. In a typical mapping application, users expect to click on a given polygon and immediately access data. Interactivity of this type is generally Windows-like behavior and is difficult to accomplish with Web-based technology where interactivity is limited. Applications and widgets exist that improve interactivity within a web environment, but they violated one of the other constraints of the project, which were maximized accessibility with no additional software purchases or downloads. Technologies such as Flex, Flash and JavaFS were options, but each of them would have created severe roadblocks to some users' ability to access the portals. The application of Ajax technology allowed for the development of a Windows-type environment and the posting of data dynamically without requiring page refresh.

Interactivity in the new portals includes dynamic user-defined queries (demographic parameters, date range of data) using sliding scales, as opposed to the canned, preset reports in the previous incarnation of the database. Also, Census tracts are dynamically highlighted as the user hovers over specific Census tract numbers in the portal navigation, allowing them to locate tracts visually as well as numerically. In the future the portals will allow users to actually drag a given polygon into a basket.

### On-the-fly calculations

The Census tracts in the data set vary widely in population density, making comparison of relative incidence rates from one to another a complex set of calculations. Those calculations take time, which then detracts from the user experience. Calculating relative radius for identified clinics was also an issue. In the Beta version, we solved both by using the ASP.NET data caching method. The next version will have the ability to save frequently used reports to minimize the amount of calculation time used by the system. The next version of the application will calculate all of the different permutations and save them in the database.

We have met and solved many challenges in developing these portals, but there are still technological challenges to be addressed. The system does not perform optimally in Internet Explorer due to coding issues within the browser. Currently, it is recommended to access the system in Google Chrome or Mozilla Firefox. Future work will attempt to address the issue with Internet Explorer to ensure a good experience for all users. Additionally, reports are only available as a new html window in the Beta system. Future versions will utilize interactive pdf technology allowing the user to interact with the report as a pdf. This will be particularly useful once Google Public Data Explorer is implemented, providing the user the capacity to animate a time series of data within a pdf document.

Currently, the portals contain data for the Houston-Galveston-Brazoria Consolidated Metropolitan Statistical Area (CMSA). To give an idea of the scale of this current implementation, the CMSA is larger than the State of New Jersey [17]. Plans are underway to expand the next version of the portals to include multi-county, regional and, eventually, statewide data. The State of Texas is the second largest in the U.S., in both geographic area and population. The unique set of challenges in the expansion is: 1) How to enroll and maintain data from clinics serving the underserved and, 2) How to allow users to quickly access local-scale data across wide geographies. In the development of the CHIS and in the Beta portal system, the authors have worked closely with the clinics that serve the underserved population in our region and provided the assistance of a community liaison, at no charge, to encourage their enrollment and help maintain the data. Clinics that serve this target population (underserved) are very busy and often under-staffed. Providing assistance in enrollment has proven helpful to their participation. Additionally, the clinics benefit from their enrollment by increasing the visibility of the clinic and attracting new patients from portal usage. However, providing a community liaison is an expensive and time intensive investment in the portal data. As we consider expanding to larger geographies, we will have to address how to efficiently enroll and maintain data for these clinics. Approaches that are being considered include region by region expansion with the use of our current liaison working directly with the local clinics and partnering with organizations that already serve and know the clinics well, such as the Breast Health Collaborative of Texas or the Lone Star Association of Clinics. Both member organizations have state-wide enrollment of our target audience and could potentially provide assistance in data entry.

In regard to the second issue, the Beta portals present spatially referenced data to a variety of potential users. They do not, however, provide a detailed explanation of the issues with utilizing spatial data such as heterogeneity and issues with rate calculation (such as the need for spatial smoothing). These are particularly important issues for a non-spatially trained user. In the case of calculated disease rates, areas with small populations (e.g., tracts) could be subject to variance instability. This means that the rates on the map may spuriously suggest differences in the underlying risk of disease [18-20]. The Beta portals also do not explain the age-adjusted rate calculation or the use of a "standard" population. The onus, at the moment, is on the user to understand how to interpret the data provided. Consequently, the immediate next development steps of the portals will include more detailed metadata, data explanations and tutorials. Additionally, a frequently asked questions (FAQ) section will be added to address some of these issues. While these are important issues to consider, our purpose with the development of the Beta site was an attempt to create a functional local-scale "query on the fly" system for accessing a variety of public health data, not to address all the issues surrounding the use of spatial data. Future versions may also employ spatial smoothing techniques to deal with issues in presenting disease rates for small areas. Another important issue to consider in the presentation of data in an online mapping portal is the modifiable areal unit problem (MAUP). The MAUP causes data analyzed at either a varying aggregation or scale to result in different answers [7]. This is an issue that the authors can only control to a certain extent. Much of the data used in the system is natively aggregated (Census data, incidence and mortality data by tract). In the current Beta system, we only present data at the unit of aggregation it was provided to the authors in. In other words, we have made no changes to the data (aggregation or disaggregation). However, as we expand the system to allow users to access data across multiple scales, the MAUP will continue to be an important issue to consider. One way to address this is to control the scale at which data is presented, as we do currently. As part of the future work on the system, the authors plan to convene an advisory panel comprised of spatial data experts to help determine how to address this issue and many others in future versions of the system.

In the first two months following the launch of the portals, there were 395 visits by 285 unique visitors. It is difficult to know if these numbers reflect a good outcome or not. The Beta system was not marketed or advertised. It also doesn't require a login, so we were not able to record information on the users. A login will be included in the full version of the system and will facilitate better tracking of users and allow for evaluation of the system. The development of the Beta portals took a large amount of resources (both time and money). While online mapping systems, such as the portals, have the potential to reach a large number of users, the amount of resources required to develop and maintain such a system is an important consideration. Future work will be focused on evaluation and assessment of the utility of the system and its ability to meet its objectives and the needs of users. Once the system moves from Beta to the full version, a focus on marketing and training will occur.

## Conclusions

To the author's knowledge, the BHP and PSN are the first local-scale interactive online mapping interface for public health data. The uniqueness of the system lies in the amount of flexibility and control it provides to the user, while providing local-scale data. Rather than pre-categorizing data, the system gives the user the ability to create custom queries specific to their research needs at local-scale geography. For users, this is a critical advancement. The ability to easily access data (for example, breast cancer mortality in African American women aged 40-64 years) at a local geography is essential to conducting research within specific geographies or specific populations. The authors feel that the major contribution of this work is in serving as an example of what can be done using the latest technologies and serving as a launching platform for others to improve on this work. With that in mind, the authors would encourage all interested researchers to contact us if they have an interest in developing similar systems or if they are interested in partnering on continued developments to our site. Initial feedback for the system has been encouraging, particularly regarding the level of user control.

## Methods

A series of focus groups were held with a cross-section of local researchers to gain insight into how people used the previous version of CHIS and how they would utilize the new system. Organizations participating in the focus groups from across the region included representatives from city and county public health officials, Federally Qualified Health Centers (FQHCs), the Harris County Healthcare Alliance (HCHA) and university-based academic researchers. Participants were asked to provide information about what kind of data they use, how they access it, how they use it and what kind of system would be useful for their research needs. Based on participant feedback and suggestions, initial development of the next generation of interactive mapping portals began. The Breast Health Portal (BHP), focused on breast cancer and access to mammography screening, and the Project Safety Net (PSN), focused on access to primary health care services. The first phase included mock-ups of the system, followed by a prototype (beta website). Feedback on these mock-ups was sought from community (researchers) throughout the development process.

### Database Development

**Figure 4:**
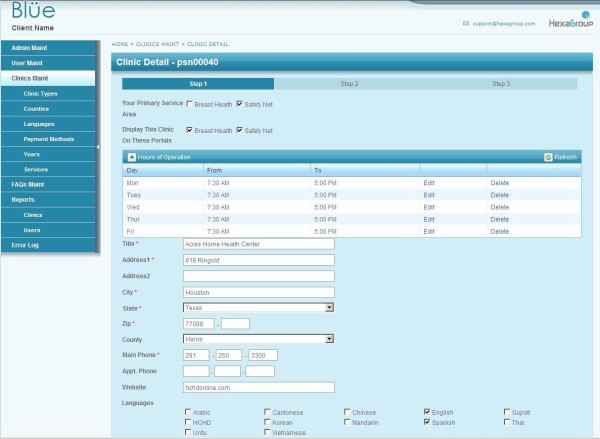
**Data entry screen for clinics participating on the portals**.

The Beta mapping portals utilize a sequel server (SQL) database to store all data. In the development of the Beta system, the previous CHIS databases (n = 12) were consolidated into a single SQL database. This was done to streamline data queries and data entry. Data entry for the portals in the Beta system is separated into two areas: data entered directly by the clinics and all other data (e.g., incidence, mortality from the State). To facilitate data entry for the clinics, an online interface was developed that allows each clinic to rapidly enter their information into the system (Figure 4). At each clinic, a staff member is responsible for data entry and is provided a user identification and password to access the system. For clinics that need assistance, a community liaison is available to assist with data entry and answer questions that arise. The community liaison's assistance is provided at no charge to the users. Data entry for all other data types is handled by the authors and is done by uploading spreadsheets into the SQL database. Data entry for the clinics is reviewed by the community liaison prior to pushing live on the system. All other data entry goes live immediately upon entry. All data is checked prior to uploading.

### System Architecture

**Figure 5:**
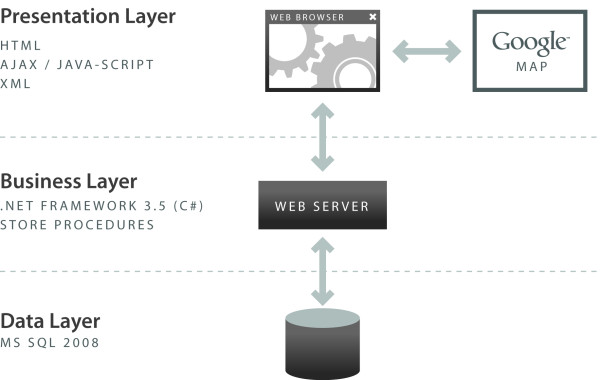
**System architecture context**.

The system offers an n-tier architecture as shown in Figure 5: Data Layer: MS SQL Server 2008, Business Layer: Store procedures and ASP.Net 3.5 Framework (C# programming), Presentation Layer: Html, Cascade Style Sheet and JavaScript (jQuery Framework). The technology used for the portals include: Microsoft .NET Framework 3.5, Microsoft SQL Server 2008, Google Maps Javascript API V3, JQuery, Telerik RadControls (ASP.NET AJAX), XML and HTML. Telerik RadControls and JQuery are used as part of the presentation layer of the application to enhance the user experience. The data used in the portals were originally ArcGIS shapefiles containing spatial and attribute data. The shapefiles were converted into KMZ (a KML compressed file), then to KML using ESRI ArcInfo. The KML files were imported into and used by Google to make the online maps. The KML spatial data retained sufficient information to "join" data from the data tables (in SQL). The data were joined using FIPS (Federal Information Processing Standards) codes. The spatial data were interactively rendered by Google upon request using HTML. Required hardware included windows based servers (Minimum 1 database server and one web server. The platform is load balancing compatible and can scale to high traffic).

**Figure 6:**
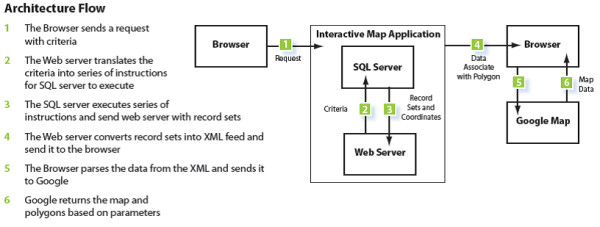
**System architecture flow**.

The application flow is as follows: the user sends a request via his/her web browser, including his/her selection criteria, which the web server translates into a series of instructions for the SQL Server to execute. The SQL Server executes a series of instructions and sends the record sets to the Web server. The Web server converts the record sets into an XML feed prior to sending it to the Web browser. The browser parses the data from the XML and sends it to Google, which then returns a map with a series of layered polygons and markers based on the user's selected parameters (Figure 6).

The polygons returned by the Google mapping API are expressions of coordinates that the user sees as either specific Census tracts or Zip code areas. They are essentially shaped containers of the proprietary information housed in the database, which is then overlayed on top of Google's generic map information. In the Breast Health Portal, all polygons are expressions of Census tracts. In the Project Safety Net Portal, information is available for both Census tracts and Zip codes. Both the polygons are shaded and the legend is dynamically created based on the user's specific search parameters. All other data on the portals (cancer incidence and mortality, birth, uninsured, etc) are attributed to a specific Census tract or Zip code. The data is received pre-categorized at these geographies. The system also generates polygon outlines expressing a variety of political boundaries: School District, Commissioner District, Senate and House districts, Counties, etc. The markers are represented as colored squares display health centers locations, type, clinic name, contact information and services offered.

C# coding language was used for programming the system. SQL Server 2008 was used for data storage. The database has been designed to allow for easy data updates, without requiring the assistance of a database administrator. New data can be imported in .csv format and allow for updating the content of the portals. The Beta system contained over 11 GB of data comprised of 52 tables and 59 store procedures used to produce the maps.

### Geographic coverage and data

**Figure 7:**
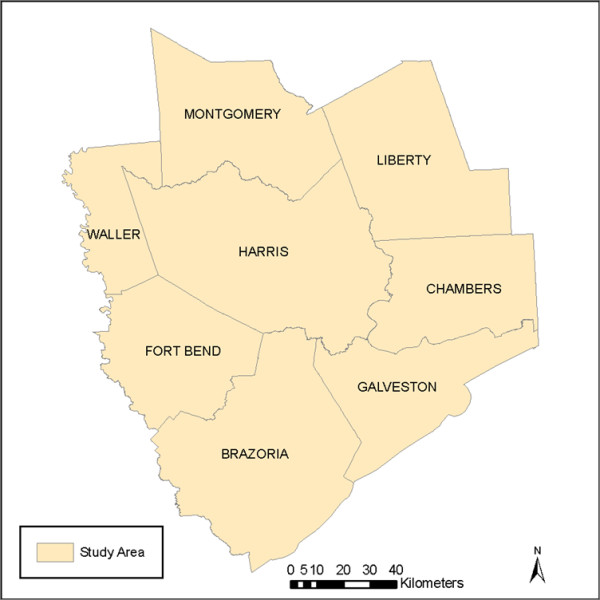
**Greater Houston CMSA Study Region**.

Geographic coverage for both Beta portals was the Houston-Galveston-Brazoria Consolidated Metropolitan Statistical Area (CMSA). The CMSA is comprised of eight counties: Brazoria, Chambers, Fort Bend, Galveston, Harris, Liberty, Montgomery and Waller (Figure 7) [17]. The CMSA covers a geographical area of 8,778 square miles (30,108 km2) and is the sixth largest MSA in the U.S., with a population of just under 6 million [17]. The population is centered in the city of Houston, with a population of just over 2 million [17].

Data available on the BHP include: age, race, uninsured status, poverty, cancer incidence (1995-2007), and mortality rates (2000-2006) over single or multiple years. In addition, point locations of service providers are included. . Additionally, geo-political boundaries such as school districts, city council, and neighborhoods can be overlaid to create customized and comprehensive maps. Data on PSN include: age, race, uninsured status, poverty, birth rates, death rates for the top five leading causes of death as defined by the Centers for Disease Control and Prevention (CDC) and medically underserved areas, in addition to point locations of service providers. Multiple sources provided data: cancer incidence data from the Texas Cancer Registry (Texas Department of State Health Services), death data from Texas Vital Statistics, clinic locations for free and low-cost health services from local clinics registered on the site, preventable emergency department visits from the University of Texas School of Public Health, medically underserved areas from the Health Research Services Administration (HRSA) and mammogram capacity as reported by all agencies offering low-cost mammography screening in the study region. Data on cancer incidence and mortality were calculated as rates per 100,000, age-adjusted to the U.S. standard population, 2000. Under IRB agreement, cancer incidence rates could only be calculated for tracts with five or more cases.

The BHP allows users to access data at Census tract geography. The PSN allows users to access data at from both Census tract or Zip code geographies and toggle between the two. Both portals allow the user to overlay geographic boundaries on selected data, such as school districts, congressional districts and neighborhoods (tract or Zip code) and to generate customized reports based on user selections.

### Usability

Both the mapping interface and provider search rely on up-to-date clinic information. The maintenance of this part of the database is managed by a community liaison. The first generation of the database required IT involvement when generating reports, adding new clinics, or authorizing administrative privileges. The second generation now has a backend that is much easier to use, streamlining the process for all involved. This is achieved by relying on Ajax based controls and improved layout of field data, which has accelerated data input by almost 50%. Information upload is significantly faster with less time eaten by data refresh. Clinics can jump from step to step/section to section with much greater ease (Figure 4). They are also able to retrieve username and passwords via email as opposed to the rigorous process they once had to endure. For the liaison, administration of the clinic information is greatly improved: The administrator can now create custom reports and add clinics, and assign users and passwords without going through the IT department. The administrator can also dynamically create new fields and tables for site scalability (clinic types, counties, languages, payment methods, years, services.) They can also easily move through the various sections of the application, which has been reported as a vast improvement over the previously cumbersome backend of the first generation.

**Figure 8:**
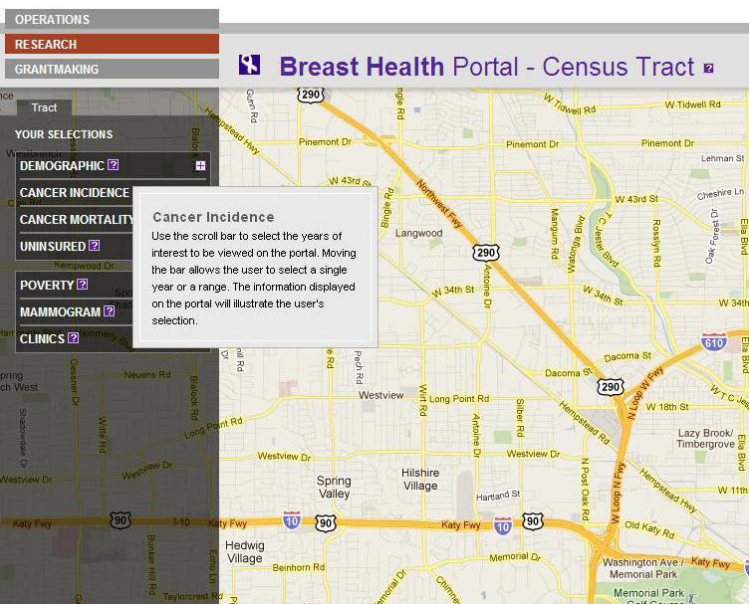
**Example of usability in the Breast Health Portal**.

On the front end of the portals, users interact with the portals via left-side navigation containing a series of expandable characteristics, all of which can be queried (for example, population data) by clicking either on the variable name or plus sign at the side of the variable (see Figure 1 and 2). Additionally, usability is improved by providing users a series of question marks on the interface. Each question mark may be clicked on. Once clicked a box open that contains definitions of related terms (Figure 8). Once selected, a drop down box opens containing detailed data available to query (for example, age and race) (Figure 2). The user selects the data either by using a slider bar or filling in a box to indicate their selection. The user can find all selections at the top of the left-side navigation. The system automatically queries the database and returns the results in real-time. Each time the user updates the query selections or panes the map, a new request is generated and the viewable map is updated, which is a major reason the system's loading time is manageable. The system's zoom has also been specially programmed to be limited based on browser version and type. For example, Internet Explorer (IE) 6 JavaScript engine is very poor at executing large amounts of scripting; therefore, when opened in IE 6, the portals' zooms are limited to levels that won't crash the application. After generating selected data, users could create a report showing their selected geographic locations and corresponding data as a new html window (Figure 3).

## Competing interests

The authors declare that they have no competing interests.

## Authors' contributions

LH developed the conceptual design for the Beta portals, collected and analyzed data and drafted the manuscript. CO carried out the focus groups and contributed to the draft manuscript. JA carried out the technical development and programming of the portals. AD oversaw all technical development and had final approval on the implementation. All authors read and approved the final manuscript.
